# A Critical Review of Materials Enhancing the Performance of Polymer Membranes for Membrane Distillation of Saline Water

**DOI:** 10.3390/nano16100616

**Published:** 2026-05-17

**Authors:** Nobuhle C. Nyembe, Olawumi Sadare, Michael O. Daramola, David Lokhat

**Affiliations:** 1 Discipline of Chemical Engineering, School of Engineering, University of KwaZulu-Natal, Durban 4041, South Africa; 2 Energy and Bioproducts Research Institute, College of Engineering and Physical Sciences, Aston University, Birmingham B4 7ET, UK; o.sadare@aston.ac.uk; 3 Sustainable Energy and Environment Research Group (SEERG), Department of Chemical Engineering, Faculty of Engineering, Built Environment and Information Technology (EBIT), University of Pretoria, Hatfield Campus, Pretoria 0002, South Africa; michael.daramola@up.ac.za

**Keywords:** membrane distillation, nanoparticles, desalination, salinity, membrane modification

## Abstract

Membrane distillation (MD) is an attractive complementary technology to conventional desalination systems. Yet commercial uptake remains limited by membrane pore wetting, temperature polarisation, and material trade-offs. This review critically examines polymeric membranes and demonstrates that reported performance gains cannot be attributed to individual polymers or fillers alone, but rather to optimised structure–property interactions governing wetting resistance, mass transfer, and mechanical integrity. Through a comparative analysis of benchmark metrics (water flux, contact angle, and liquid entry pressure), we identify recurring failure mechanisms, including nanoparticle agglomeration, coating instability, and hydrophobicity-driven compromises in liquid entry pressure and durability. Moving beyond a descriptive summary of materials, this review introduces a predictive structure–property–performance framework that systematically links dominant operational limitations and targeted modification strategies. The analysis reveals that surface-localised, adhesion-controlled modifications outperform bulk approaches by preserving pore architecture while mitigating fouling and wetting risks. Key research priorities include validation under high-salinity conditions relevant to brine management, standardised environmental and leaching assessments of nanomaterials, scalable fabrication protocols supported by techno-economic considerations, and developments on bioinspired materials. By shifting focus from material novelty toward rational design principles, this review establishes actionable selection criteria to accelerate the translation of MD membranes from laboratory concepts to industrially viable desalination technologies.

## 1. Introduction

Water shortage is a global challenge, with over 2 billion people of the global population living in water-stressed regions, while at least 1.7 billion people have water that does not meet the required standards [1]. Research and development efforts continue to explore new and more efficient ways to desalinate seawater, focusing on reducing energy consumption, environmental impact, and costs. Membrane technology is at the forefront of wastewater treatment advancements, offering cost-effective solutions with compact equipment, low power consumption, and high pollutant removal efficiency. Membrane distillation (MD) has attracted increasing attention as a promising desalination technology. This is due to its ability to utilise low-grade thermal energy and operate at low temperatures [2]. Bhadrachari et al. [3] demonstrated the potential of operating an MD system on a solar heater while treating reverse osmosis brine, achieving fluxes between 1.5 and 2 $\frac{L}{m^{2}\cdot h}$ (LMH). A study by Ma et al. [4] also demonstrated that solar ultra-low-grade heat (<50 °C) could be used to supply energy with the assistance of a photothermally modified membrane, which could be attractive for remote areas. Integrated into a water pyrolysis process, MD feed was used for heat recovery, serving as a cooling stream in a heat exchanger to eliminate the heating energy demand [5]. Additionally, MD has proven highly efficient in treating streams with high salinity, achieving salt rejection rates exceeding 99%.

**Figure 1:**
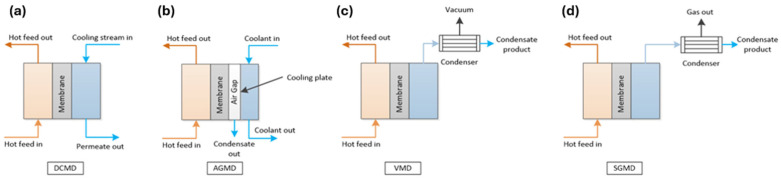
Schematic presentations of membrane distillation configurations (**a**) DCMD; (**b**) AGMD; (**c**) VMD; and (**d**) SGMD (adapted from Osman et al. [6]).

MD operates on a phase-separation principle, in which the heated feed and cold permeate flow on either side of a hydrophobic membrane. Separation is driven by the temperature gradient, which creates a vapor pressure difference across the hydrophobic porous membrane, separating the two streams. It can be implemented in four primary configurations, as shown in Figure 1: (a) direct contact membrane distillation (DCMD), (b) air-gap membrane distillation (AGMD), (c) sweep-gas membrane distillation (SGMD), and (d) vacuum membrane distillation (VMD). Each configuration differs in the way the vapor is collected and condensed.

The development of appropriate membrane materials plays a significant role in the efficiency and feasibility of MD systems. Artificial intelligence is also supporting the rapid development and optimisation of desalination technologies by predicting material performance, screening for the best materials, and enabling process control for optimum performance, thereby reducing the cost of experimental trial and error [7,8,9,10].

Despite the rapid growth of nanomaterial-enabled MD membranes, current studies report performance improvements under varying experimental conditions, including different base polymers, fabrication methods, MD configurations, and feed compositions. For example, PVDF membranes modified with SiO_2_ nanoparticles produced fluxes ranging from 2.9 to 17 LMH, either operating on a different configuration, testing different feed conditions, or using membranes prepared with different methods [11,12,13]. As a result, direct comparison between studies makes it difficult to isolate the true contribution of specific nanomaterials.

The field still lacks a unifying framework for the coupled trade-offs among wetting resistance, vapor transport, and thermal penalties, particularly because surface layers and coatings that improve hydrophobicity can simultaneously reduce vapor flux. In addition, long-term stability remains under-resolved due to key failure modes, such as nanoparticle aggregation, leaching, and fouling, which are acknowledged but rarely synthesised into actionable design rules and durability benchmarks [14,15,16,17]. Finally, translation barriers remain insufficiently addressed and cost–benefit analyses are largely absent, while environmental considerations for common nanomaterials are often treated peripherally rather than embedded into material selection.

Table 1 shows relevant review articles obtained on Scopus with the combination of search words “membrane distillation,” “materials,” “nanoparticles,” “desalination,” and “seawater” between 2019 and 2026. These reviews compare performance on heterogeneous systems (different polymers, fabrication methods, configurations, and feed sources). The current review therefore aims to address a critical gap in the MD literature by presenting a unified, nanomaterial-centric framework that systematically links membrane design, structure–property relationships, and process performance. This will be of particular importance to the research community working on membrane development as it provides benchmarks on material selection fit for the purpose. The objectives of this review are therefore to:

(i) Evaluate modification methods and nanofillers by benefits, drawbacks, and scalability.
(ii) Analyse the relationship between membrane structure, material composition, and performance metrics such as flux, contact angle, and liquid entry pressure.
(iii) Compare membrane performance under controlled conditions (base polymer, configuration, and feed concentration) to improve interpretability.
(iv) Assess bioinspired materials as emerging materials for membranes.

**Figure 2:**
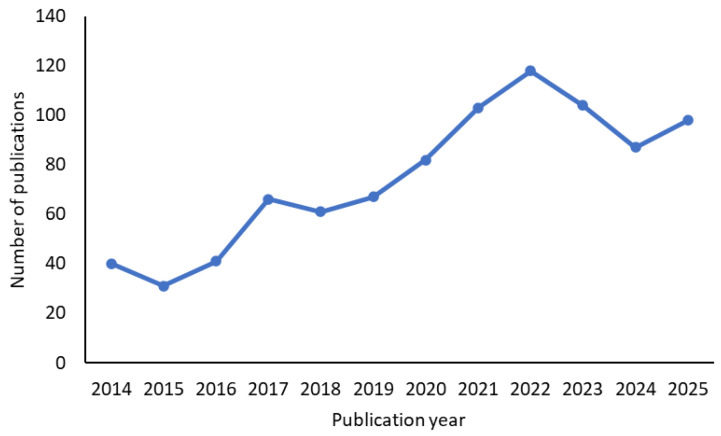
Publications between 2014 and 2025 on Scopus using keywords “membrane distillation” and “seawater desalination” as of May 2026.

This work is presented as a critical review supported by a targeted literature survey rather than a fully systematic review. Relevant studies were identified using the Scopus and Google Scholar databases. Searches were conducted using combinations of the keywords “membrane distillation,” “materials,” “nanoparticles,” “desalination,” and “seawater.” The review focused on publications from 2014 to 2025 to capture recent advances in membrane material development, identifying 1200 articles using the broader search terms “membrane distillation” and “seawater desalination,” as shown in Figure 2. Literature selection was performed through iterative screening of titles, abstracts, and full texts, with additional influential studies included based on their relevance. Studies primarily addressing equipment design, process modelling, or simulation were excluded to maintain focus on material-related aspects. In total, approximately 80–100 studies informed the analysis presented in this review.

**Table 1:** Comparison of recent membrane distillation (MD) reviews (2019–2026).

Year	Focus/Contribution	Key Strength	Limitations/Gaps
2026 (This review)	Material-centric benchmarking of nanoparticle-modified membranes; structure–property–performance relationships	Controlled comparison (same polymer/configuration/feed); introduces benchmarking metrics (flux, LEP, CA); emerging bioinspired materials for sustainability	Framework may require adaptation for different base conditions
2026 [18]	Advanced nanomembranes, MD configurations, modelling, and performance analysis	Comprehensive coverage of emerging nanomaterials (MOFs, COFs, photothermal)	Compares different polymers/configurations, which makes it difficult to isolate nanoparticle effects; limited focus on saline feed performance
2025 [19]	Porous organic & inorganic nanomaterials (MOFs, COFs, CNTs); synthesis & performance	Strong material synthesis insight; discusses cost & toxicity	Heterogeneous comparisons (different fabrication methods, feeds, configurations); lacks standardised benchmarking
2025 [20]	Electrospun nanofibre membranes (TiO_2_, SiO_2_, Al_2_O_3_) for fouling mitigation; green materials: biodegradable polymers, natural fillers, green solvents	Deep focus on electrospinning; fouling and wetting prevention; introduces sustainability in MD materials	Limited to one fabrication method; not general across MD membranes
2024 [21]	Carbon-based nanomaterials (CNTs, GO, carbon black) in MD	Detailed discussion on carbon materials and fabrication routes	Strong material bias; no selection framework; comparisons across inconsistent systems
2023 [22]	Nanomaterial-incorporated membranes (metal oxides, graphene, MOFs, quantum dots)	Broad overview of materials and fabrication techniques	No standardised comparison, performance reported under varying conditions
2023 [16]	Photothermal MD membranes (CNTs, Au, Ag)	Highlights heat-transfer enhancement mechanisms	Lacks comparative performance matrix; limited scalability discussion
2022 [23]	Superhydrophobic inorganic nanomaterials for MD desalination (graphene-based, CNTs, metal oxides, clays, zeolitic imidazole frameworks)	Focus on hydrophobicity enhancement strategies	Cost of achieving superhydrophobicity and interventions to limit fouling; no environmental considerations
2021 [24]	Metal oxides vs carbon nanomaterials in RO and MD	Cross-technology comparison; nanoparticles economic insights	Mixing technologies (RO & MD) obscures MD-specific conclusions
2020 [25]	Evaluates the incorporation of nanomaterials such as quantum dots, metalloids, metal oxide-based, MOFs & carbon-based	Covers incorporation techniques and fouling control	Inconsistent feed conditions make comparison difficult
2020 [26]	Nanotechnology applications in MD membranes	Focus on carbon-based and metal-based materials	Limited depth in MD-specific mechanisms; limited material comparison
2019 [27]	Nanoparticle-enhanced MD membranes; synthesis techniques	Cost implications of modified membranes	Comparisons across different feeds/configurations
2019 [28]	Nanomaterials in desalination (MD, RO, FO, etc.)	Multi-technology perspective	Not MD-specific; lacks detailed MD material analysis

## 2. Membrane Distillation as an Emerging Desalination Technology

Desalination technology has been adopted in most countries over the years, with a steady increase of 6.8% per year since 2010, accounting for an additional water supply of more than 4.6 million m^3^/day worldwide [29]. The most widely applied technologies include multi-stage flash (MSF), multi-effect distillation (MED), and reverse osmosis (RO), which have reached high levels of technological maturity. These are compared to MD as an emerging technology with distinct advantages and limitations in Table 2.

The wide adoption of conventional desalination technologies can be attributed to their technological maturity, large-scale operability, and well-optimised performance. Thermal processes such as MSF and MED are robust and can handle variable feed conditions, while RO has relatively lower specific energy consumption than thermal methods and has become the industry standard for seawater desalination [30]. However, the main limitation of desalination technologies is energy requirements across all desalination systems. MSF and MED require significant thermal energy input, while RO relies heavily on electricity for high-pressure pumping. Energy consumption is a serious challenge, as most countries still rely on fossil fuels, which are big contributors to greenhouse gas emissions. It has been estimated that desalination could contribute up to 0.4 billion tons of CO_2_ equivalent annually by 2050, highlighting the urgency of integrating renewable energy sources [31,32]. MD offers a competitive advantage in this context due to its ability to operate at lower temperatures (typically 40–80 °C), with ongoing research exploring the use of low-grade heat. Additionally, MD is less sensitive to feed salinity and can, in theory, achieve near-complete rejection of non-volatile solutes [16]. These features make MD particularly attractive for treating hypersaline brines where RO becomes inefficient or infeasible.

Despite its advantages, MD systems generally exhibit lower permeate flux than RO, which limits productivity and increases the required membrane area. In contrast, RO systems have undergone decades of optimisation, including the development of energy recovery devices and advanced membrane materials, making them significantly more energy-efficient at scale. Therefore, rather than serving as a direct replacement for established technologies, MD is increasingly being positioned as a complementary or hybrid solution [33]. For instance, RO has recovery rates between 35 and 55%, where at 40% recovery rates, the salinity of the produced brine is about 1.66 times higher than that of seawater [34]. Beyond such limits, MD could be more beneficial for increasing productivity as a complementary system.

Economically, conventional technologies currently maintain a clear advantage due to established infrastructure, economies of scale, and optimised supply chains. In contrast, MD is still at a developmental stage, with higher membrane costs, lower flux, and limited large-scale demonstration.

**Table 2:** Comparison of membrane distillation to conventional desalination technologies.

Technology	Advantages	Disadvantages	Salinity Limit (mg/L) [35]	Capacity (103 m^3^/day) [30]	Water Cost (USD/m^3^) [30]	Operational Cost (USD/m^3^) [36]	Energy Consumption (kWh/m^3^) [30]
MSF	High-quality product Commercialised Scalable	High capital cost Requires a large land footprint Equipment corrosion High energy requirements Total shutdown for maintenance	No limit	50–70	0.52–1.5	1.5–2.5	13.5–25.5
MED				0.6–30	0.56–1.75	1.0–2.0	6.5–11
RO	Operates at low temperatures Flexible compact design Commercialised	High operating pressures Membrane fouling Expensive membranes Low recovery rates Brine production Expensive chemicals	≤60,000	1–320	0.45–1.72	0.5–1.0	3–6
MD [33,37]	Can treat highly saline streams Can use low grade heat Ambient operating pressures High salt rejections	Low fluxes High energy requirements Limited large-scale demonstration	≤100,000	-	-	3.2–3.7	22–67

## 3. Membrane Materials

According to the literature, an ideal MD membrane should exhibit hydrophobicity, a porous structure, a well-suited distribution of pore size, high liquid entry pressure, high fouling resistance, and excellent mechanical strength [38,39]. Membrane materials that are conventionally used in MD processes for seawater desalination include polyvinylidene fluoride (PVDF), polytetrafluoroethylene (PTFE), polypropylene (PP), polyethylene (PE), and hydrophobic ceramic membranes, as presented in Table 3 [40,41,42].

PVDF is a versatile synthetic polymer valued for its high thermal stability (decomposing above 400 °C) and resistance to surface wetting. However, its hydrophobicity can be unstable under MD operating conditions, often resulting in low vapor flux and increased susceptibility to fouling [43,44,45]. In contrast, PTFE (Teflon) is highly hydrophobic, chemically inert, and temperature-resistant, making it effective for MD. Despite these advantages, its practical deployment is constrained by poor solubility and high crystallinity, which necessitate complex fabrication techniques such as sintering and stretching [23].

PP offers a more economical alternative and has been explored for MD primarily due to its low cost and ease of processing. Nevertheless, PP membranes generally exhibit lower hydrophobicity and inferior thermal and chemical resistance compared to PVDF and PTFE, which can limit their long-term performance [23]. Beyond polymeric materials, ceramic membranes based on alumina, titania, and zirconia have been investigated for desalination owing to their excellent mechanical strength, thermal stability, and chemical robustness. However, their intrinsic brittleness and hydrophilic nature necessitate extensive surface modification to prevent pore wetting, thereby increasing cost and complexity and ultimately restricting their widespread application in MD [40,46,47].

**Figure 3:**
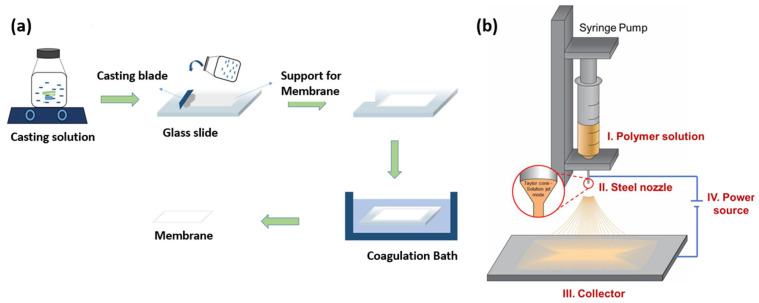
Schematic representation of the (**a**) phase inversion process [51] and (**b**) electrospinning process [52].

Polymeric membranes are widely used in MD technology primarily due to their inherent hydrophobicity and ease of processing [16]. However, across base polymers, increased intrinsic hydrophobicity (e.g., PTFE) rarely translates into superior techno-economics because fabrication costs and limited scalability counterbalance performance gains. Most of the available commercial membranes used in MD are not specifically for MD application but are originally for microfiltration or ultrafiltration; hence, they are more prone to low permeability and pore wetting [38,48]. These commercial membranes are often available as flat sheets or tubular PTFE, PVDF, and PP materials [49]. They are commonly fabricated by phase inversion and electrospinning (Figure 3), which influences their structure, morphology, and performance, whilst PTFE is fabricated by mechanical biaxial stretching, spinning, auxiliary-assisted pore forming, or paste extrusion [50].

PVDF remains the most practical platform for membrane distillation (MD) research due to its ease of processing, chemical stability, and morphological tunability. However, unmodified PVDF membranes are frequently only marginally hydrophobic (water contact angle ≈ 90°), placing them near the wetting threshold. As a result, their wetting resistance can deteriorate during operation, particularly under low-surface-tension conditions (e.g., in the presence of alcohols or surfactants), where pore intrusion and flux collapse have been reported [53]. In contrast, modifying PVDF to produce superhydrophobic surfaces (WCA ≥ 150–160°) significantly enhances anti-wetting behaviour and can substantially increase the liquid entry pressure (e.g., from ~0.58 to 3.38 bar), thereby conferring markedly improved resistance to wetting challenges and operational instability [54].

The limitations of polymeric membranes, particularly wetting, fouling, thermal stability, and scalability, are reported in the literature as the critical constraints on membrane distillation performance and long-term operation. Polymeric membranes tend to have an increasing moisture content over time, which exacerbates the resistance to mass transfer for vapor molecule diffusion across the membrane [55]. This phenomenon is called membrane pore wetting. This process is governed by the liquid entry pressure (LEP), defined as the minimum pressure required for liquid to penetrate membrane pores, and should typically be above 2.5 bar [23,56]. Hydrophilic membranes exhibit low LEP and are therefore more prone to wetting at relatively low pressures. To mitigate this, membranes should be highly hydrophobic, with larger contact angles, to maintain higher LEP and resist pore wetting. However, hydrophobic membranes also suffer from fouling and delayed wetting. This implies that membrane modification should balance surface hydrophobicity and mass transfer. These challenges have consequently driven the development of advanced material modification strategies, which form the central focus of this review.

## 4. Physical Membrane Surface Modification

Nanomaterial-modified membranes have emerged as a promising approach to overcome the limitations of base polymer membranes, enhancing hydrophobicity, water flux, fouling resistance, and surface properties. Nanoparticles can be incorporated onto membrane surfaces using various physical techniques, such as spraying, dip coating, and layer-by-layer assembly [57]. These methods enable the attachment or immobilisation of nanoparticles to enhance membrane properties.

### 4.1. Dip and Spray Coating

**Figure 4:**
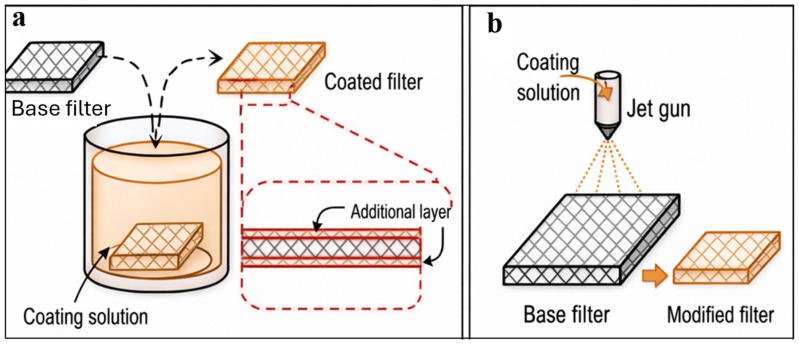
Schematic illustration of membrane surface modification by (**a**) dip coating and (**b**) spray coating techniques [58].

The spraying and dip coating methods shown in Figure 4 are targeted at modifying the surface charge of the polymer membrane, which is often linked to limiting fouling and pore wetting. In dip coating, the membrane is immersed in a coating solution and withdrawn at a controlled rate, forming a uniform surface layer after drying or curing. In spray coating, the coating solution is atomised and deposited onto the membrane surface, enabling controlled layer thickness and localised surface modification. These methods have the advantage of retaining the properties of the polymer membrane.

In preventing pore wetting while aiming for enhanced flux, MD membranes will contain a highly porous sublayer and a microporous hydrophobic top layer [59]. This can be achieved by spray coating, in which one side of the membrane, typically the active layer, is modified without altering the opposite side. Hydrophilic–hydrophobic composite membranes, achieved by spray-coating a hydrophilic layer onto a hydrophobic membrane surface, have been shown to resist wetting. This was observed in a study by Wang et al. [60], where they developed a superhydrophilic–hydrophobic composite membrane by coating a PVDF membrane with perfluorooctanoate (for antifouling), chitosan (for hydrophilicity) and silicon nanoparticles (for surface roughness). The coating improved the membrane contact angle from 105° to 133°, producing a flux of about 25 LMH while maintaining a salt rejection of >99.9% over 20 h of operation, indicating the absence of pore wetting and fouling.

Membrane surface dip coating with nanoparticles, such as carbon nanotubes (CNTs), graphene, and metal–organic frameworks (MOFs), enhances membrane permeability and salt rejection [61]. Covalent bonds facilitate the bonding of coat monomers to the polymer chains through chemical, radiation, photochemical, and plasma-induced techniques [62]. Membrane surface coating, however, is limited by the potential for the coating solution to block membrane pores, further increasing permeation resistance [63]. Modifying hydrophobic membranes can be challenging due to the instability of some nanoparticles and the polymer membrane. Nevertheless, using polyelectrolytes provides a uniform coating and strong adhesion to the membrane surface, thereby significantly improving the membrane’s stability and performance [64].

### 4.2. Layering Coating

**Figure 5:**
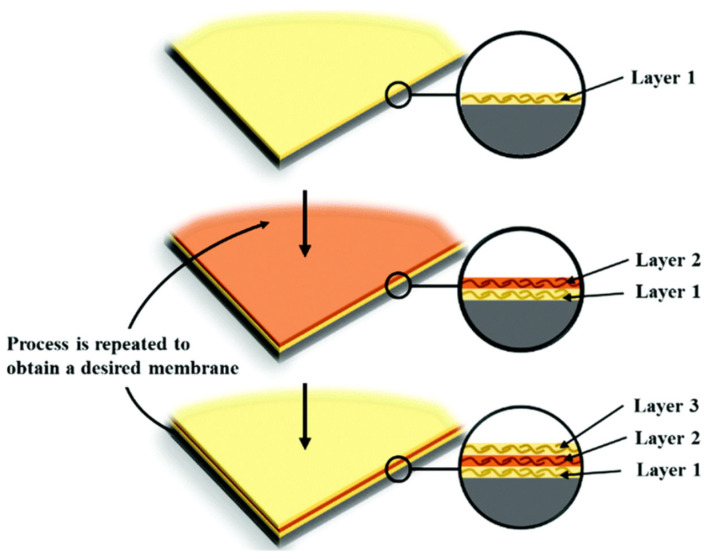
Schematic diagram of the layering casting method [67].

To address the membrane-wetting challenge, layer-by-layer coating has enabled the development of multiple-layer membranes, as shown in Figure 5. This is achieved by integrating multiple coating layers during fabrication to form double- or triple-layered membranes. Hydrophobic/hydrophilic double-layer membranes have a thin hydrophobic side facing the hot stream as a selective layer and prevent pore wetting, while the thick hydrophilic side faces the permeate stream and provides support [65]. A double-layer membrane was prepared by Mat Radzi and Ahmad [66], who cast PVDF–HF on top of PVDF. However, PVDF has demonstrated limited compatibility with other hydrophilic polymers during phase separation, resulting in a decrease in the mechanical strength of the membranes [63]. Polymer blending should, however, be practiced with caution, as the interaction between blended polymers has the potential to induce instability in both kinetic and thermodynamic properties [63].

### 4.3. Coating Limitations

The choice of technique often involves a trade-off between precise control and pore integrity/stability. Layering provides exceptional nanoscale control over coating thickness and chemistry; however, the sequential adsorption of the coat often leads to pore infiltration and internal film growth, particularly when low-molecular-weight polymers are used or when coating is performed in situ. This pore constriction can significantly reduce effective porosity and vapor flux, while the predominantly electrostatic interactions render layering films vulnerable to high salinity, extreme pH, and oxidising environments, limiting their durability under realistic desalination conditions. Stacking of coating layers introduces increased transport resistance, as observed in a study by Ray et al. [68], where superhydrophobicity was achieved at the expense of pore narrowing and increased membrane thickness, highlighting the need for alternative strategies that improve surface functionality without compromising pore accessibility or interfacial stability.

Dip coating, while attractive due to its simplicity, offers limited control over coating thickness and spatial localisation. Capillary forces during membrane immersion and withdrawal promote uncontrolled solution intrusion into membrane pores, leading to pore blocking, especially when viscous or highly concentrated coating solutions are used. Moreover, adhesion between the coating and the substrate is highly dependent on weak physical interactions, making dip-coated layers susceptible to swelling, delamination, and leaching during prolonged exposure to thermal and chemical stresses.

Spray coating has emerged as a compromise between precision and practicality, as it promotes more surface-localised deposition and reduces bulk pore infiltration compared to dip coating. This was observed in a study by Garcez et al. [69], where increasing the poly(dimethylsiloxane) (PDMS) concentration during PVDF dip coating led to a 28–43% reduction in pore size, indicating pore blocking, whereas spray coating only resulted in a 4% reduction in pore size, implying minimal impact. Nonetheless, achieving uniform coverage remains challenging, particularly on highly porous or rough membrane surfaces. Non-uniform droplet distribution and overspray can lead to inhomogeneous coating thickness, creating mechanically weak regions that act as failure initiation sites during long-term operation. To mitigate such challenges, Elahi et al. [70] incorporated cyclic olefin polymer into the coating solution to increase the mechanical strength, stability of nanoparticle immobilisation, and adhesion of the coating to the PP substrate.

To overcome these limitations, recent research has increasingly focused on hybrid and chemically anchored strategies, including spray-assisted LbL assembly to limit pore infiltration, cross-linking of LbL films to enhance chemical stability, and covalent grafting or bio-inspired adhesive interlayers (e.g., polydopamine) to improve coating adhesion [71]. In preventing pore blockage during dip coating, Talebi et al. [72] investigated filling membrane pores with ethanol, 2-propanol, or glycerol to prevent the infiltration of the coating solution into the membrane pores. These approaches aim to decouple surface functionalisation from pore structure modification, thereby preserving membrane permeability while enhancing coating durability under harsh membrane distillation conditions. Given the importance of preserving membrane porosity while ensuring durability, Table 4 compares these conventional coating techniques with respect to pore-blocking tendency and long-term stability, scalability, and costs.

Kwon et al. [73] distinguish between small- and large-scale membrane surface modification methods by emphasising that scalability should not be assessed solely by equipment size or throughput, but rather by the ability to achieve consistent, reproducible coating performance. From this perspective, batch processes such as dip coating are inherently limited, as maintaining uniform coating thickness and penetration across multiple cycles is challenging. Consequently, while dip coating is simple and widely used at the laboratory scale, its practical scalability is constrained by reproducibility issues. In contrast, more continuous approaches such as spray coating and layering offer improved control over deposition and are more amenable to consistent large-area processing, making them more suitable for industrial-scale implementation. Dip coating is generally considered a low-cost technique due to its simple equipment requirements and ease of operation. In contrast, spray coating typically incurs moderate costs, as it requires additional control systems (e.g., for droplet size, flow rate, and deposition conditions) to ensure uniform coating. However, the overall cost advantage of dip coating can diminish at larger scales, where increased solvent consumption and material waste arising from bulk immersion and limited coating efficiency can significantly elevate operational expenses [74].

## 5. Nanomaterial Internal Structure Modification

Membrane surface coating produces thin-film composite (TFC) membranes, whereas blending nanoparticles into the membrane matrix yields mixed-matrix membranes (MMMs) [62]. Mixed matrix membranes (MMMs) are composed of an organic polymer serving as the continuous phase and a filler acting as the dispersed phase. Thin-film composite membranes consist of a thin, highly selective layer and a stable support [75]. The modification of membranes by integrating nanoparticles into the polymer matrix is a strategy to combat membrane fouling and enhance hydrophobicity, thereby boosting flux rates [23,76,77]. Performance improvements have been observed through reduced membrane pore wetting and enhanced anti-fouling when using nanoparticle infusion and/or surface coating [78,79,80]. The importance of nanoparticles is that they alter membrane porosity by filling macro-voids, thereby increasing the liquid entry pressure and thus reducing pore wetting. Furthermore, they introduce a level of membrane surface roughness. This is important in MD, as the use of rough membranes promotes feed turbulence, which in turn reduces boundary-layer resistance to heat transfer and enhances the efficiency of vapor transport [81]. Several nanoparticles have been investigated for integration into polymer membranes for MD applications in seawater treatment, including carbon-based, metal-based, zeolite, and recently bioinspired materials.

### 5.1. Carbon-Based Nanomaterials

Organic nanoparticles, such as the carbon-based variants, are well known for their exceptional selectivity, resistance to fouling, compatibility with biological systems, cost-effective production, lightweight nature, and flexibility [61]. These materials, such as graphene, carbon nanotubes (CNTs), and activated carbon (AC), are renowned for their hydrophobic characteristics and effective adsorption/desorption capabilities [38].

#### 5.1.1. Graphene

Graphene and its derivatives, such as graphene oxide, offer unique properties that make them promising materials for a range of innovative applications. This is because it has several functional groups, which also makes it easier to attach new functional groups through functionalisation [82,83]. In membrane technology, graphene-based membranes exhibit exceptional capabilities in water and gas purification [84,85]. In the context of water treatment, graphene oxide is more common due to the strong attraction of water to the oxidised parts of the GO’s surface and edges, as well as the swift, smooth flow of water over the flat graphene regions, making it have high water permeation rates [82]. With a surface area of about 2600 m^2^/g and an absorption rate of up to 277 g/g, this means that graphene oxide can absorb a significant amount of water relative to its weight [83].

Recent advancements in graphene-based membranes for filtration purposes, highlighting monolayer nano-porous graphene membranes, graphene oxide membranes, and polymeric membranes incorporating graphene oxide, are emerging as promising candidates [41]. However, in MD, graphene is disadvantaged by the high thermal conductivity and poor interaction of the nanosheets with polymers, resulting in a limited exploration of their benefits [86]. An augmentation in both surface and internal membrane porosity was achieved alongside an improvement in the average pore size when Leaper et al. [82] investigated the incorporation of 3-(aminopropyl) triethoxysilane-functionalised graphene oxide into PVDF, resulting in an 86% increase in permeate flux. The presence of the silane functional group improved the dispersion of the graphene nanoparticles by forming covalent bonds, thereby creating a barrier that repels them from one another. This repulsion helped prevent agglomeration and promoted better dispersion of the nanoparticles in the medium.

The hydrophilic GO has been considered for MD membrane modification, as its processing is cheaper compared to pure graphene [83]. However, this also presents challenges of reduced flux due to the limited fraction of GO that can be used in a polymer [86]. Mao et al. [86] developed a novel porous and hydrophobic GO-based PVDF membrane for the treatment of 35 g/L NaCl saline water using DCMD to investigate the water vapor transport behaviour in the GO/polymer membrane matrix. With the assistance of polydimethylsilane (PDMS) for hydrophobicity and metal–organic framework-801 (MOF-801) for porosity, the MOF-801@GO-1%PDMS/PVDF membrane depicted an enhanced flux of 15–29 kg/m^2^.h with no reduction in hydrophobicity and limited pore wetting over 72 h of operation. In addition to this, a life cycle analysis showed that incorporating GO into polymer membranes could reduce energy consumption by 27–34% for MD membranes due to increased permeability [87]. According to Mao et al. [86], there are limited efforts in the development of hydrophobic GO membranes to improve the vapor transport in MD. This can be attributed to the cost and the additional modification required to make GO hydrophobic.

#### 5.1.2. Carbon Nanotubes (CNTs)

CNTs offer a range of desirable properties and benefits that make them valuable in various applications, particularly in the field of desalination and membrane technology. They are nanostructures composed of one-atom-thick, rolled carbon sheets, forming elongated, hollow cylindrical structures with diameters ranging from 1 nm to several centimetres. Their strong covalent bonding between carbon atoms, which are naturally held together by van der Waals forces, offers them exceptional strength. The remarkable properties of CNTs, including their adsorptive qualities, strength and low weight, ease of functionalisation, and large surface area, make them promising materials for a wide array of desalination applications [88].

Incorporating CNTs into membrane matrices results in substantial improvements in permeability, solute rejection, and tensile strength, as well as a decrease in fouling tendencies, making them invaluable assets in membrane technology for a variety of applications [89]. A PVDF membrane infused with fluorinated CNTs showed an improvement in LEP from 273 kPa to 500 kPa, indicating enhanced mechanical strength, reduced pore size, and increased water contact angle [78]. The reduced porosity can be attributed to an increase in viscosity of the PVDF polymer solution in the presence of CNTs, leading to a reduced exchange rate of solvent and non-solvent during phase inversion [45]. The downside of CNTs is the costs associated with their synthesis, long-term operation, and production scale-up [16,61]. The high thermal conductivity of CNTs, however, limits their application in MD membranes, as it promotes heat transfer, leading to higher heat loss. In addition to this, there is concern about the potential release of CNTs into treated water [23].

Multi-walled CNTs (MWCNTs) have been well demonstrated as effective additives in a PVDF composite membrane, where the contact angle increased from 82° to 87° while maintaining the membrane surface porosity, due to the strong interaction between the NPs and the polymer [89]. The study also showed that CNTs should be infused with caution, as they tend to form aggregates at higher concentrations, leading to increased membrane pore size and reduced mechanical strength. Nevertheless, the dispersion of CNTs can be improved by tailoring the sequential mixing protocol for polymer, CNTs, and solvent [90]. A study by Zhou et al. [89] investigated the impact of adding MWCNTs and silicon dioxide nanoparticles (SiO_2_ NPs) to PVDF composite membranes for vapor membrane distillation. When MWCNTs were added, they increased membrane porosity and the number of macro-voids, leading to higher flux but reduced mechanical properties. The highest flux was achieved at 25 °C and 50 °C with 2 wt.% MWCNTs and salt rejection exceeding 99.98%. They then added SiO_2_ NPs at low ratios to MWCNTs. This synergistically enhanced membrane performance.

#### 5.1.3. Activated Carbon (AC)

Hydrophobic porous activated carbon (AC) is characterised by multi-directional internal channels that promote vapor transport to enhance flux [49]. Zhao et al. [38] reported that AC nanoparticles possess a strong water vapor adsorption/desorption capacity due to their high surface area and porosity. When incorporated into a PVDF–HFP membrane, these nanoparticles modified the membrane’s structure and surface properties, enhancing vapor transport pathways and facilitating more efficient transfer of water vapor through the pores. This structural enhancement was reflected in an increase in water vapor flux from 36.4 LMH to 45.6 LMH. Further, the AC-incorporated membrane maintained a high flux and salt rejection rate (99.5%) even under a concentrated solution (10 wt.%), which can be an indication of minimal pore wetting. However, like most nanoparticles, AC is also not exempt from the challenges of particle aggregation. Functionalisation using fluoroalkyl groups has been studied to reduce particle aggregation and promote dispersion in membrane matrices [49].

#### 5.1.4. Carbon-Based Nanomaterials Performance Comparison

Table 5 presents a comparison of membrane distillation (MD) membranes modified with various carbon-based nanoparticles. The data reveal a clear trend toward membranes with higher surface contact angles, indicating greater hydrophobicity in the presence of the modified nanoparticles and consistently improved water vapor flux. This correlation underscores the critical role of hydrophobicity in MD performance, as it prevents pore wetting and enhances vapor transport through the membrane.

This suggests that the introduction of hydrophobic carbon-based nanomaterials not only enhances the membrane’s resistance to wetting but also optimises its permeability. This trend is attributed to nanoparticles’ ability to create additional vapor-transport pathways while maintaining structural integrity. However, the extent of improvement varies depending on the type of nanomaterial and its dispersion within the polymer matrix. For example, while CNTs significantly boost mechanical strength and solute rejection, their tendency to aggregate at higher concentrations can offset gains in flux [78]. Conversely, membranes incorporating hydrophilic fillers may exhibit high water flux, but often suffer from poor salt rejection due to pore wetting [86]. These observations highlight the importance of balancing hydrophobicity with other membrane properties, such as porosity and thermal conductivity, to achieve optimal MD performance. A study by Sabet [91] shows that carbon-based nanoparticle functionalisation can improve dispersibility and adhesion on the polymer membrane, preventing nanoparticle agglomeration and leaching while maintaining their functionality. Additional precautions like the use of surfactants and optimisation of sonication time are recommended to improve dispersibility, while advanced functionalisation techniques are recommended to control the density and distribution of functional groups.

### 5.2. Metal-Based and Oxide Nanoparticles

Metals such as silver, gold, and copper, as well as metal oxides like silica oxide, titanium dioxide, and zinc oxide, have been used to modify membranes.

#### 5.2.1. Silver Nanoparticles (AgNPs)

One of the challenges in membrane technology is fouling, characterised by the deposition, attachment, and proliferation of organic and microbial contaminants on the membrane surface, leading to reduced performance over time. This consequently impacts the operational and environmental costs of the process by elevating hydraulic pressure resistance and accelerating the membrane’s performance deterioration. Silver nanoparticles (AgNPs) have been identified as an effective antibacterial and antifungal filler to combat the biofouling challenge, as they can disrupt bacterial cell membranes and interfere with cellular processes [92]. However, they have the potential to contaminate the surrounding environment due to their tendency to leach from the membrane during use.

Research efforts have been directed towards mitigating or decreasing the rate at which silver ions are released from membranes. Various methods have been explored, such as enhancing the silver content, creating silver composites, carrying out functionalisation, or incorporating nanoparticles into the membrane polymer rather than applying them as a coat [92]. Mpala et al. [45] used cellulose nanocrystals to minimise Ag NP leaching by immobilising them. The presence of nanoparticles improved the contact angle of a PVDF membrane from 82.6° to 92.9° and increased the liquid entry pressure (LEP) from 310 kPa to 640 kPa, while maintaining a minimum leaching of 0.378 ppm Ag over time. However, the permeate flux decreased from 0.53 LMH to 0.179 LMH, attributed to pore blockage by AgNPs.

A study conducted by Vafaei et al. [56] demonstrated that applying a polydopamine (PDA) coating to PES facilitates the attachment of silver nanoparticles (AgNP) modified with 1-dodecanethiol. This modification was found to effectively limit leaching of AgNPs, as evidenced by an undetectable Ag concentration in the permeate and by maintaining a CA of 95° from 109° after chemical treatment of the membranes. The PDA further immobilises the Ag nanoparticles [93]. Compared with other nanoparticles, the use of AgNPs offers benefits for CA and LEP improvement but comes at the cost of reduced permeate flux. Sulfidation is recommended to strike a balance between minimal leaching and biofouling resistance, but it also risks diminished antibacterial efficacy with excessive treatment [92]. Meanwhile, the PDA coating demonstrates superior leaching resistance, albeit with limited long-term performance data. Each method thus presents trade-offs between membrane performance parameters and operational longevity, necessitating further research to optimise silver retention while maintaining efficacy.

#### 5.2.2. Titanium Nanoparticles (TiNPs)

Titanium dioxide nanoparticles have also been used as antibacterial and antifouling fillers. However, they have the potential to reduce a membrane’s hydrophobicity due to the hydroxyl group [94]. Hence, the functionalisation of TiO_2_ NP by fluorosilane to improve the membrane’s hydrophobicity, reduce membrane fouling, and enhance the membrane’s antibacterial resistance has been investigated. A PVDF membrane loaded with silane–TiO_2_ NP had an improved contact angle (131.7°) and reduced biofouling, especially towards E. coli [94].

Improved thermal efficiency, reducing membrane temperature polarisation, was observed when TiNPs were used as a coat to enhance the performance of a PVDF membrane in solar AGMD [95]. This was attributed to the interconnected, porous, hydrophilic TiNP coating, evidenced by the drastic reduction in the membrane’s surface contact angle, which facilitated enhanced mass and heat transfer. Salt rejections of ~99.9% were obtained despite the increased hydrophilicity, which could enhance membrane pore wetting. In a similar study, two types of TiNPs (hydrophilic and hydrophobic) were investigated [96]. The hydrophobic TiNPs increased the membrane’s LEP, while the hydrophilic TiNPs increased the membrane’s flux, risking pore wetting. The fusion of these particles achieved a balanced midpoint with an average LEP of 207 kPa and an improved flux of 4.4 LMH. A PVDF membrane incorporating TiO_2_ and silane-modified nanocellulose nanoparticles achieved improved porosity, TiNP dispersion, and hydrophobicity, enabling the treatment of 100 g/L NaCl brine with a flux of 2 LMH and 99.4% salt rejection [34].

#### 5.2.3. Zinc Nanoparticles

Zinc oxide nanoparticles (ZnO NPs) have properties such as non-toxicity, high thermal conductivity, photochemical activity, and antibacterial activity [66]. In MD, these properties enhance membrane performance by inhibiting bacterial growth and biofouling on the membrane surface, degrading organic contaminants in the feed, and improving heat transfer across the membrane. Mat Radzi and Ahmad [66] investigated the effect of ZnO NPs in a dual-layer PVDF membrane when treating seawater from a fish farm (38 g/L NaCl) using DCMD operated over 360 h. The incorporation of ZnO NPs increased the membrane’s top-layer hydrophobicity from 136° to 140°. However, there was an agglomeration of NPs at higher loadings (2%), which could compromise the mechanical strength of the membrane. Furthermore, the ZnO NPs reduced the membrane LEP from 0.69 to 0.4 bar, thereby exposing the membrane to pore wetting. Nevertheless, the membrane achieved 99.9% salt rejection at a flux of 9.42 LMH.

In another study investigating the treatment of a 12–13 g/L NaCl solution using DCMD, a PVDF membrane incorporated with amine-functionalised ZnO NPs was used [97]. This membrane displayed an increase in permeate flux (from 16 to 25 LMH) at 0.5 wt% ZnO loading. Increasing the ZnO NPs loading beyond this point resulted in reduced membrane porosity, ultimately reducing membrane flux. This can be attributed to the occlusion of membrane pores as a result of poor NP dispersion. The presence of ZnO in the produced membranes has been shown to improve flux, selectivity, and fouling resistance in membrane distillation applications. However, it is essential to optimise the concentration and dispersion of ZnO NPs to ensure their effective integration into the membrane structure without compromising its mechanical strength or stability.

#### 5.2.4. Other Metals and Metal Oxide Nanoparticles

Other metal oxide nanoparticles investigated in MD include, but are not limited to, copper (CuNPs), calcium (CaNPs), cobalt (CoNPs), iron (FeNPs), and aluminium (AlNPs). The abundant copper nanoparticles have been used as anti-biofouling substances in membranes for desalination [98]. Copper nanoparticles offer the benefit of being more cost-effective to manufacture. In a study by Shahlol et al. [99], CuO and Al_2_O_3_ showed a reduction in the water contact angle of a polysulfone/polyethylene glycol membrane, which was attributed to the presence of oxides, which make the membrane more hydrophilic as they form hydrogen bonds with water. Saldias et al. [98] developed hydrophobic CuO-NPs functionalised with a thiol group, which improved PVDF CA and reduced pore size. However, the membrane produced low flux (3.9 LMH) when tested in a solution with very low salinity (0.1 wt.% NaCl); thus, this membrane required further modification by coating to reduce its surface energy.

In a study by Song et al. [100], the presence of CaCO_3_ in PVDF improved its permeability and porous structure at a dosage of less than 2 wt.%, while increasing beyond this point resulted in a reduction in porosity, which was attributed to nanoparticle aggregation that weakened the pore structure and contributed to the formation of closed pores, also reducing the membrane’s tensile strength. Similar results were obtained in another study; however, a reduction in LEP was also observed [101]. In a comparative study on hydrophilic CuO and CaCO_3_, CuO emerged as more effective in PVDF membrane modification, as it demonstrated improved membrane performance, attributed to its capacity to augment membrane structure by enhancing its porosity [102]. Cobalt oxide nanoparticles modified by oleic acid were used to enhance the hydrophobicity of a PES membranes and improve the dispersion of nanoparticles in the PES polymer solution [103]. The membrane with a maximum loading of 4 wt.% nanoparticles was able to improve the hydrophobicity (CA = 106°) and was able to maintain a water flux of 9.5–11.6 LMH for 120 min. At increased feed concentrations, however, this membrane had reduced permeate flux.

Iron and its oxides have been explored for their magnetic and photothermal properties in membrane technology. Magnetite finds application in MD due to its ability to improve membrane microstructure and prevent fouling, as was shown in a study by Agbaje et al. [59] where they explored magnetite as a hydrophilic enhancer of a PVDF membrane active surface, which was found to increase flux significantly.

Metal–organic frameworks (MOFs) are a class of materials constructed from metal ions or clusters bonded to organic ligands to form one-, two-, or three-dimensional structures. They exhibit high surface areas, ordered tuneable nanopores, low thermal conductivity, low density (0.2–1 g/cm^3^), and diverse chemical functionalities, making them attractive for various applications [104,105]. However, the utilisation of MOFs in water treatment is constrained due to the structural degradation experienced by certain MOFs when exposed to aqueous environments [105]. Aluminium fumarate (AlFu) MOF is one of the MOFs that have shown reasonable stability and was incorporated into PVDF, tested on a DCDM membrane [104]. The AlFu MOF was able to enhance its surface roughness and improve the pore structure, thereby establishing sufficient vapor pathways, resulting in a notable flux of 22 LMH while maintaining stable operating flux throughout 46 h of real seawater treatment. Aluminium, however, demonstrates high thermal conductivity, which contributes to temperature polarisation in DCMD; hence, in some instances, membranes containing aluminium might require coating with thermal insulating material.

#### 5.2.5. Silica Oxide Nanoparticles (SiNPs)

Silica oxide nanoparticles are also incorporated into the membrane material to enhance the membrane’s properties, such as hydrophobicity, mechanical strength, and thermal stability. SiNPs exhibit traits that enhance membrane flux by enlarging macro-voids, yet this enhancement comes at the expense of weakening the membrane’s mechanical integrity and decreasing its LEP, thereby enhancing membrane wettability [89]. They can, however, improve the thermal stability of the membrane, allowing it to withstand the high temperatures often encountered in membrane distillation processes. This was observed in a polycarbonate/silica aerogel NP mixed matrix membrane, which demonstrated an increased thermal stability due to the increased membrane thickness, reducing the membrane heat loss [106].

The mechanical strength of a PVDF membrane was reduced from 1.07 MPa to 0.39 MPa in the presence of SiO_2_ nanoparticles, due to the formation of macro-voids, which cause structural defects [78]. However, the presence of SiO_2_ nanoparticles in this membrane was able to improve water flux from 11.36 LMH to 19.88 LMH while maintaining a salt rejection rate of >99%. Similarly, in a study by Efome et al. [11], silica nanoparticles were blended with PVDF polymer for the casting of PVDF/SiO_2_ membrane at varying SiO_2_ composition (1–10 wt.%). The superhydrophobic nanoparticles induced uneven water distribution during membrane fabrication, leading to localised water accumulation and larger pore sizes, thereby increasing water flux from 0.75 LMH to 2.9 LMH at a 7 wt.% loading; however, their presence also reduced the membrane’s LEP from 490 kPa psi to 414–434 kPa.

In a study by Bose et al. [63], to address the challenge of silicon carbide nanoparticle agglomeration, they introduced functionalisation with the amine group using 3-aminopropyl (diethoxy) methyl silane. Mistry et al. [12] took it a step further and prepared superhydrophobic modified silica nanoparticles with a contact angle of 151° and a stable dispersion. This was achieved by reacting the silica nanoparticles with octadecyl trichlorosilane in toluene. Although the nanoparticles were superhydrophobic, this hydrophobicity did not transfer well when incorporated into PVDF, as the contact angle only increased to 92° from 73°. Furthermore, the introduction of nanoparticles increased the LEP from 324 to 330 kPa at 5.3 wt.% silica loading, indicating a very slight reduction in membrane pore wetting potential. Nevertheless, this membrane achieved better performance at lower nanoparticle loading.

Similar to other metal oxide NPs, SiNPs can compromise the mechanical strength of a polymer; hence, their inclusion should be moderate and used in conjunction with mechanical strength-enhancing additives such as MWCNTs [23,89]. SiO_2_ was blended with MWCNTs in a PVDF composite membrane for the treatment of 35 g/L NaCl saline water using VMD, increasing the flux from 1.25 to 4.55 LMH without reducing the LEP, hence limiting membrane pore wetting [89]. Nthunya et al. [107] took it a step further and modified SiO_2_ nanoparticles with silane reagents to form superhydrophobic SiO_2_ nanoparticles. This increased the membrane’s CA from 96° to 160°. To increase membrane fouling resistance, they coated the formed PVDF/SiO_2_ membrane with a hydrophilic thin layer containing MWCNTs and AgNPs, which was able to sustain a constant flux and salt rejection over 50 h of operation until 24–37% flux reduction was observed due to the onset of fouling, with a slight reduction in salt rejection. This indicates a promising approach to mitigating fouling and pore wetting. Although promising, a downside of this study is the reduction in flux from 43 LMH to 17 LMH.

Fluoroalkyl functionalisation also showed potential to improve SiNPs’ hydrophobicity, which resulted in reduced surface energy of a PVDF membrane when the fluorinated SiNPs were used as a coat [108]. This coat maintained the membrane’s porosity but increased its CA from 95° to 164°, resulting in a 3.4-fold increase in flux from 4.1 to 14.3 LMH. These studies show great potential for the application of SiNPs; however, the propensity of membrane fouling is not investigated in detail. Furthermore, they are investigated using synthetic water, where their performance is uncertain in the presence of competing ions. In addition, SiNP dispersion within the membrane matrix and their interactions with other membrane components must be carefully controlled to ensure optimal performance and stability of the membrane in membrane distillation applications.

#### 5.2.6. Metal—Based and Oxide Nanoparticles Performance Comparison

Whilst each nanoparticle filler offers unique advantages, their successful integration into polymer membranes requires careful optimisation of loading, functionalisation, and composite design. As illustrated in Table 6, silicon nanoparticles (SiNPs) demonstrate superior hydrophobicity but suffer from LEP reduction, compromising membranes’ mechanical strength. Nevertheless, according to Vafei et al. [56], MD membranes require an LEP greater than 2.5 bars to resist wetting. In contrast, AgNPs exhibit remarkable flux enhancement but are prone to leaching. Though many fillers increase flux and/or CA, LEP responses are non-monotonic and filler-loading-sensitive, highlighting wetting risk at higher loadings (e.g., AgNP-PES LEP drop).

These comparative data underscore that nanoparticle selection must align with specific operational priorities, whether fouling resistance, flux maximisation, or mechanical stability, while accounting for trade-offs in leaching, agglomeration, and production costs. Table 7 highlights the key benefits and drawbacks of some metal-based and oxide nanoparticles. It is evident that their properties complement each other; hence, hybrid approaches, such as SiO_2_-MWCNT composites or PDA-coated AgNPs, are promising strategies to mitigate the limitations of individual materials. However, as demonstrated in various studies, achieving optimal performance requires careful control of nanoparticle loading to preserve membrane integrity. They all require verification under natural seawater matrices with organic matter and scaling precursors.

### 5.3. Zeolites

Zeolites are crystalline aluminosilicate minerals characterised by a three-dimensional framework structure consisting of aluminium, silicon, and oxygen atoms. The structure, formed by the arrangement of zeolite tetrahedra (TO4; T = Si, Al) sharing oxygen atoms, creates tunnel-like passages that form cavities suitable for accommodating water or metal ions during filtration [110]. They have a porous structure with regularly spaced channels and cages, making them useful in various industrial applications. One of their distinctive characteristics is their capacity for both adsorption and desorption of molecules, along with a high surface area, hydrothermal stability, well-defined microporosity, and a uniform pore structure and size [111]. There are numerous types of zeolites, each with a specific structure and properties, and they are named based on their mineral composition. The Si/Al ratio plays a crucial role in determining the type of zeolite that is formed. A high Si/Al ratio primarily results in the formation of hydrophobic zeolite [110]. A distinctive characteristic of zeolites is that they do not swell, and they can withstand very high temperatures. They are effective at removing various contaminants, such as heavy metal ions and organic and inorganic contaminants, due to their small pore size [112].

The hydrophilic Linde Type-A (LTA) zeolite features micropores with a diameter of 0.4 nm, making it a suitable candidate for molecular sieving separation [113,114,115]. The infusion of this nanoparticle results in a smooth surface of the membrane, creating molecular hydrophilic channels that enhance water permeability and a negatively charged surface, which ensures salt ion rejection [88]. This implies that LTA might be a challenge for MD, as it allows water molecules to pass, risking membrane pore wetting. Nevertheless, the pore size of LTA restricts the permeation of the hydrated ions (Na^+^ (0.716 nm) and Cl^-^ (0.664 nm)) present in saline water [115]. Zeolites have also been used as adsorbents in adsorption desalination due to their porous properties, which enable them to absorb water vapor [116]. Linde type-L (LTL) used as a coat for poly(vinylidene fluoride-co-hexafluoropropylene) was able to minimise fouling and enhance flux while maintaining a high LEP [117]. This was attributed to a reduced pore size and porosity in the polymer, which restricted the permeability of liquid water. Ammonium mordenite zeolites were also used as an adsorbent suspended in synthetic seawater to remove salinity (Na) and hardness (Ca and Mg) for a VMD system [118]. The presence of this zeolite improved membrane flux, which was sustained for a prolonged period due to the removal of scaling agents, thereby minimising membrane fouling.

A hydrophobic Zeolitic Imidazolate Framework (ZIF) with a pore size of 0.34 nm and properties such as chemical stability, thermal stability, uniform micropores, and high surface area has also been investigated for MD application [115]. In a study by Salehi et al. [119], ZIF-8 was modified through the introduction of ZnO nanoparticles and a silane coupling agent. Integrated into a PVDF polymer, this was tested on a DCMD configuration for seawater desalination. The presence of the nanoparticles improved hydrophobicity and porosity; however, the LEP decreased, suggesting that the membrane is more susceptible to pore wetting. Furthermore, the modified membranes showed negative flux in some instances, attributed to insufficient temperature difference, which was compensated by the osmotic pressure difference between the streams.

Taking advantage of the hydrophobic ZIF-8 and hydrophilic chitosan, Kebria et al. [75] fabricated PVDF/ZIF-8/chitosan membranes for the treatment of seawater. The presence of the chitosan coat reduced the membrane’s pore size while increasing its porosity, thereby increasing LEP. It further increased membrane flux by three-fold while minimising fouling. Another hydrophobic zeolite is zeolite Y, with an Si/Al = 5, which was able to improve PES hydrophobicity from a CA of 138° to 160° by coating [120].

Zeolites offer a promising solution for improving the efficiency and sustainability of water treatment processes through their unique properties and versatile applications in membrane technology. It should be noted that their application as membrane fillers for MD is limited because they are often hydrophilic. Nevertheless, MMMs with dispersed zeolites in the polymer solution can be further modified by surface coating to enhance their hydrophobicity [121]. An important limitation of zeolites in membranes is their high cost, difficult reproducibility, and aggregation of nanoparticles, which results in membrane defects.

### 5.4. Decision Flow

**Figure 6:**
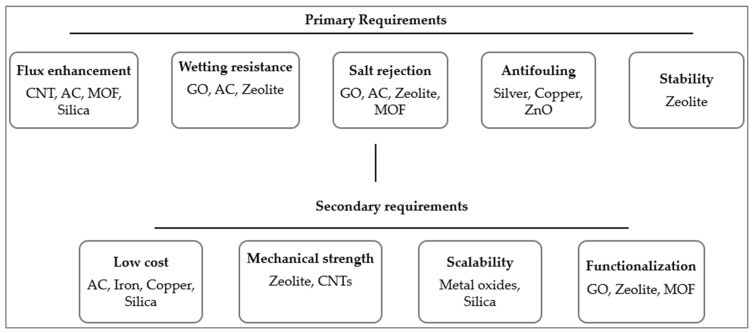
Decision flow diagram on selecting nanoparticles based on the required membrane improvement.

Selection of nanoparticle modifiers for membrane distillation (MD) membranes is highly application-specific and requires balancing multiple, often competing, performance criteria. For PVDF membranes treating saline streams, the primary challenges are sustaining high vapor flux while mitigating severe inorganic scaling, without compromising wetting resistance and long-term stability. The reviewed studies have shown that nanoparticles can beneficially tailor membrane structure, surface chemistry, and interfacial interactions; however, inappropriate selection, loading, or incorporation routes can lead to pore blockage, increased mass-transfer resistance, nanoparticle agglomeration, or accelerated pore wetting. To rationalise the nanoparticle choice, a decision-flow framework is proposed in Figure 6.

The framework systematically links dominant performance limitations and modifiers to guide the selection of suitable nanoparticle families for PVDF membranes. Since PVDF membranes often struggle to sustain long-term MD operation due to insufficient intrinsic hydrophobicity, the selection of nanomaterials should aim to address multiple primary performance requirements simultaneously. An effective strategy would be to engineer stable, low-surface-energy interfaces while preserving the membrane’s intrinsic pore structure and porosity. Following the proposed decision framework, zeolites emerge as promising candidates because they meet several primary requirements for MD membranes, including wetting resistance, salt rejection, and structural stability. Their well-defined crystalline pore architecture can enhance selective transport and improve mechanical and thermal robustness under harsh operating conditions. However, they may require surface functionalisation to improve compatibility and dispersion in the polymer matrix. Furthermore, the modified membrane would require surface coating to preserve the internal structure. Similarly, if the aim is to produce membranes at a large scale, metal oxides are better candidates; however, a secondary approach should be adopted to ensure membrane stability and to achieve the other primary requirements.

This approach enables the development of a modified membrane with a balanced solution. Generally, zeolites are best deployed within thin, coated matrices or paired with hydrophobic top layers. Hydrophobic MOFs can be blended at modest loadings, provided they are suitably functionalised to demonstrate stability in saline water. When budget and reproducibility dominate, fluorinated silica topcoats can be preferable, whereas when flux stability against wetting is paramount and cost is secondary, carbon-based materials could be attractive, subject to coating for stability.

## 6. Bioinspired Membrane Modification

Biodegradable nanoparticles ensure the membrane material is environmentally friendly and can degrade into harmless by-products after use. This is crucial for sustainability and reducing environmental impact. Many biodegradable materials are abundant and inexpensive, making them cost-effective for large-scale production of membranes. According to Sirohi et al. [61], depending on fabrication conditions and other factors, biodegradable nanoparticles can be obtained from renewable resources such as cellulose, polylactic acid, chitosan, starch, alginate, and gelatine.

Chitosan, sourced from chitin in crustacean shells like crabs and lobsters, is a biopolymer boasting biocompatibility, hydrophilicity, antibacterial qualities, and excellent film-forming abilities [75]. The presence of the nitro (N & O) functional group ($C_{6}H_{11}NO_{4}X_{2})$ promotes the attraction of water molecules [75]. In a study by Elizalde [48], a PVDF/chitosan membrane evaluated in an AGMD configuration showed large pore sizes, resulting in pore wetting and reduced salt rejection, attributed to the hydrophilic nature of chitosan. Despite this, chitosan binds easily to polymers such as PVDF due to its amine groups and has a good absorption capacity for metals due to the presence of the amino and hydroxyl groups [122]. Hence, it has been integrated with metal oxide nanoparticles to improve membrane performance [75,122]. In addition to this, chitosan has presented good antibacterial and low-fouling properties in membrane operations [62].

The widely available nanocellulose is a renewable, biodegradable material derived from cellulose fibres, which are naturally produced by biomass such as algae, bacteria, and sea creatures, as well as agricultural products [123]. Nanocellulose has good water dispersibility and a large surface area, and the presence of alcoholic hydroxyl groups makes hydrophilic cellulose easy to functionalise for membrane applications [124]. Incorporating modified nanocellulose into membrane materials can improve their hydrophobicity and mechanical strength for membrane distillation [125]. In a study by Lalia et al. [126], nanocrystalline cellulose incorporated into a PVDF-co-hexafluoropropylene matrix demonstrated improved mechanical strength and reduced the membrane pore-size distribution, resulting in an increased LEP (131 to 186 kPa) and minimising membrane pore wetting.

A study by Tan et al. [125] also demonstrated an improved membrane flux in the presence of cellulose and TiO_2_ NPs with silane membrane surface functionalisation when treating highly concentrated brine (100 g/L NaCl) in a DCMD configuration. According to review studies [123,127], nanocellulose has been mostly applied in RO, NF, MF, and pervaporation for desalination, with very limited application in MD. However, due to their hydrophilic nature, they can be useful for membrane coating to improve the membrane flux, where one side of the cellulose should be modified to be hydrophobic [127]. Furthermore, the hydrophilic nature of most bioinspired nanoparticles facilitates surface functionalisation, promoting strong interfacial interactions with polymer matrices [128].

Biodegradable nanoparticles can also serve as carriers for additives such as antimicrobial agents or fouling inhibitors. These additives can improve the performance and longevity of the membrane without causing environmental harm. Maximising on the properties of the abundant biopolymer, cellulose nanocrystals capped with AgNPs were used to modify the PVDF membrane to improve its performance while treating seawater [45]. This modification reduced pore wetting of the membrane, as evidenced by reduced membrane porosity (70%) and increased liquid entry pressure (330 kPa). However, the presence of these nanoparticles drastically reduced the permeate flux from 0.53 LMH to 0.18 LMH.

Polyhydroxyalkanoate (PHA) nanoparticles, a group of biodegradable polymers produced by diverse microorganisms from renewable waste materials, have wide applications in biomedicine for their capacity to form antiviral/antimicrobial coatings and films [129]. Further, they are non-toxic, insoluble in water, and available at an industrial scale, making them good candidates for membrane technology [130]. In membrane technology, PHA has been applied as a biopolymer due to its bacterial rejection [131,132]. However, findings on PHA-enhanced membranes in seawater indicated that membranes experience some weight loss over time [132]. This was attributed to the presence of PHA-degrading microorganisms in seawater, which also affects the membrane surface morphology. At the time of writing, no article on the use of PHA for water treatment in MD was found. According to Read et al. [133], some PHAs exhibit characteristics similar to those of polypropylene (PP). Therefore, they are worth exploring for application in membrane distillation.

Polydopamine (PDA), an environmentally friendly biopolymer, has been identified as an optimal coating material for improving MD flux, resistance to fouling and wetting, and establishing a robust foundation for subsequent modifications to the membrane surface [63]. A PVDF modified with polydimethylsiloxane and polydopamine was tested in hypersaline water (100 g/L NaCl) using a VMD configuration [134]. The presence of this coat enhanced membrane hydrophobicity (CA = 162.5°) and LEP (287 kPa). The membrane demonstrated stable, high flux (~13 LMH) and maintained salt rejections above 99.9%, indicating minimum fouling and pore wetting. An added advantage of polydopamine is the strong adhesion to various substrates, including polymeric membranes, which helps to ensure the stability and durability of the modified membranes during MD processes. However, there are limits to its chemical stability and to the synthesis process, which may use toxic solvents.

### 6.1. Bioinspired Materials Performance Comparison

Each biodegradable nanoparticle has distinct advantages and trade-offs, as outlined in Table 8. For large-scale sustainable MD applications, nanocellulose (with hydrophobic modification) appears to be the most balanced choice, whereas PDA could dominate if eco-friendly synthesis is achieved. Future research should focus on hybrid modifications that combine the strengths of different nanoparticles. For instance, integrating hydrophobically modified nanocellulose with PDA or embedding metal–organic frameworks (MOFs) or silica nanoparticles into a biodegradable matrix could enhance both membrane performance and environmental sustainability.

Compared to the metal/carbon/zeolite-based materials, these biodegradable fillers could reduce fossil-derived burden but currently face durability penalties in extended MD operation. Their life-cycle assessment advantages can, however, be eroded if biodegradation occurs in seawater microbiomes, rendering the membranes ineffective in the long term.

### 6.2. Bioinspired Material Biodegradability

**Figure 7:**
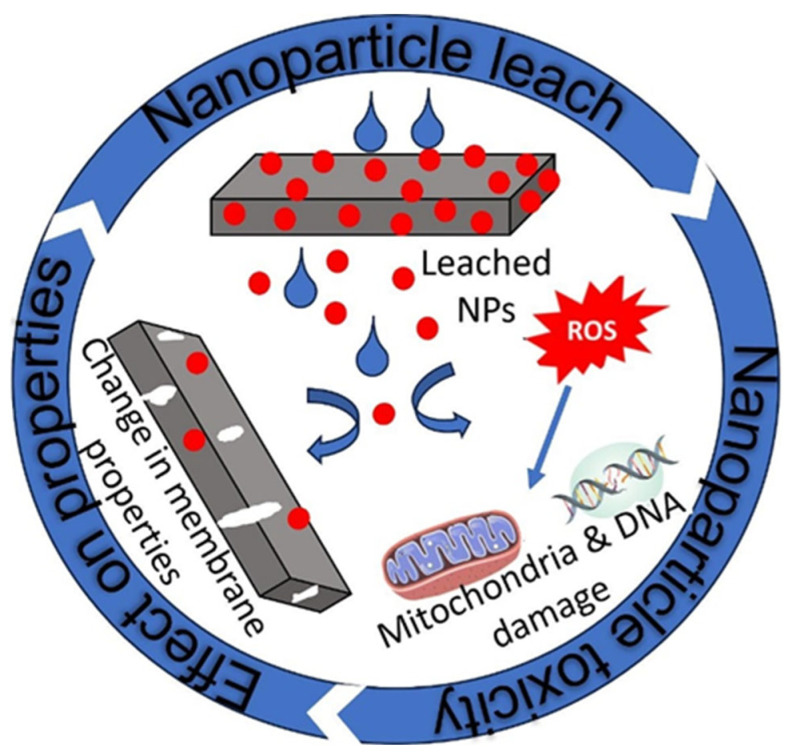
Schematic representation of the impact of nanoparticle leaching on the membrane properties and the marine ecosystem, reproduced from [137] with permission from Elsevier.

The reviewed studies have shown that some nanoparticles tend to leach from the polymer matrix during operation due to poor compatibility or the harsh conditions of the aquatic environment, leading to loss of membrane functionality and contamination of the marine ecosystem, as shown in Figure 7. This challenge can be addressed through surface functionalisation strategies that enhance nanoparticle immobilisation within the polymer matrix. For example, recent work has demonstrated that introducing functional groups (e.g., amine or carboxyl groups via plasma modification) significantly improves the attachment of biobased nanoparticles such as lignin to polymeric substrates via electrostatic interactions, hydrogen bonding, or covalent linkage [135]. To preserve the biodegradability and environmental benefits of these materials, however, eco-friendly functionalisation agents should be considered. It is worth noting that there are a few studies on the stability and implications of releasing nanoparticles into the marine environment due to the marine ecosystem complexity [136]. Metals and carbon-based nanoparticles are non-biodegradable, so their presence is known to persist for long periods in the environment.

The assumption that biologically derived materials are intrinsically environmentally safe can be misleading, particularly under marine conditions where high salinity, microbial activity, and complex organic matrices can alter nanoparticle stability, degradation pathways, and ecological interactions. Materials such as PHAs have shown biodegradation ranging from four months to two years in aquatic environments [133]. A study investigating PDA/hydroxyapatite/chitosan for wood coating indicated that it preserves structural integrity and maintains crystallinity following extended marine immersion (five months), thereby enhancing resistance to biodegradation and oxidative damage [138].

While these studies confirm the resistance of biodegradable materials in saline water, targeted research focused on membrane-specific performance is essential to enable their development as viable membrane materials. Current membrane research using bioinspired materials largely emphasises flux enhancement and fouling mitigation, whereas biodegradation behaviour, microbiome-level impacts, and regulatory compliance considerations are rarely integrated into material design.

## 7. Concluding Remarks

This review critically evaluated recent advances in membrane distillation for saline water treatment, with particular emphasis on PVDF-based membranes due to their favourable chemical resistance, processability, and widespread application. The literature consistently demonstrates that performance improvements are not dictated by any single nanofiller, but rather by the strategic integration of materials and methods that collectively mitigate wetting and fouling while maintaining liquid entry pressure (LEP) and mechanical integrity. Despite these advances, several fundamental challenges remain unresolved. A key limitation lies in the trade-off between hydrophobicity and permeability, where modifications that enhance anti-wetting properties often compromise vapor transport or structural stability. In addition, nanoparticle incorporation introduces persistent issues, including agglomeration, interfacial incompatibility, and potential leaching, raising concerns about long-term stability and environmental safety. Fouling under realistic feed conditions in high-salinity, multicomponent systems remains poorly understood. Furthermore, the lack of standardised testing protocols and performance benchmarks makes it difficult to compare results across studies and assess technological readiness. Critically, most reported studies remain at laboratory scale, with limited attention to scalability, reproducibility, and economic feasibility.

Future research should focus on the following identified areas to bridge current knowledge gaps and accelerate the practical advancement of membrane distillation technologies.

(i) Evaluate membranes in high-salinity water, where membrane distillation exhibits distinct operational advantages for brine management and zero-liquid discharge applications. Demonstrate coating durability and physicochemical stability under realistic seawater environments, accounting for complex fouling agents, multivalent ions, and long-term operational stresses.
(ii) Implement dispersion and adhesion protocols for nanoparticles to prevent agglomeration and/or leaching by introducing covalent bonding through nanoparticle surface functionalisation and combine with low-surface-energy coatings to enhance liquid entry pressure (LEP) without significantly compromising vapor flux.
(iii) Address scale-up challenges by establishing reproducible fabrication protocols, using low-cost and abundant nanomaterials, ensuring techno-economic feasibility, and developing one-step modification techniques to reduce processing complexity and energy consumption to facilitate industrial translation.
(iv) Establish standardised performance metrics and benchmarking protocols to enable meaningful comparison with established technologies such as reverse osmosis.
(v) Integrate standardised environmental evaluation frameworks, including leaching assessments and biodegradation paths, to support safety and sustainability.
(vi) Explore bioinspired functionalisation agents to modify environmentally friendly and cost-effective membranes.

Overall, MD shows strong potential for desalination, and ongoing innovations are key to its commercial viability.

## Figures and Tables

**Table 3:** Comparative benchmarks for base polymers.

Polymer	Typical Contact Angle (°)	Strengths	Limits
PVDF	90–120	Flexible Tuneable morphology Thermal stability	Wetting under extended runs Fouling
PTFE	120	Chemical resistant Thermal stability	Complex processing Fouling Cost
PP	98	Inexpensive	Mechanical degradation Low thermal resistance Low chemical resistance
Ceramics	-	Thermal stability Chemical resistant Robust	Brittle Needs coating Expensive

**Table 4:** Comparative limitations of membrane coating techniques.

Technique	Pore Blocking Tendency	Long-Term Stability	Scalability/Industrial Application	Cost
Dip coating	High—uncontrolled solution intrusion into pores during immersion and withdrawal, especially with viscous coatings	Poor to moderate—strongly dependent on substrate–coat chemistry interactions	Batch operation—simple process, widely scalable, but limited control over coating uniformity	Low–moderate
Spray coating	Moderate—predominantly surface-localised, but overspray and droplet coalescence can clog pores	Moderate—non-uniform coverage creates mechanically weak regions	Large continuous scale– compatible with large-area and roll-to-roll processing	Moderate–high
Layering	Very high—polyelectrolyte infiltration leads to internal pore growth	Conditional—electrostatic bonding is sensitive to salinity, pH, and oxidants	Large continuous scale—precise but time-intensive, limiting large-scale deployment	Moderate–high

**Table 5:** Comparative performance data for carbon-based fillers in PVDF membranes showing performance parameters before → after modification in DCMD treating ~35 g/L NaCl feed.

Filler	Membrane Preparation Method	CA (°)	Flux (LMH)	Ref.
CNT, CNC, & AgNP	Phase inversion	91.1 → 92.9	0.53 → 0.179	[45]
CNTs & SiO_2_	Phase inversion	116 → 103	7.58 → 19.88	[78]
GO	Vacuum-assisted self-assembly	38.6 → 115	104 → 29.24	[86]
AC	Phase inversion	139 → 143	36.4 → 45.6	[38]
AC	Phase inversion	92 → 133	26.5 → 40.4	[49]

CA: contact angle.

**Table 6:** Performance comparison of metal/oxide fillers in polymer membranes on DCMD configuration (pristine → modified membrane).

Filler	Polymer	Feed (g/L NaCl)	CA (°)	Flux (LMH)	LEP (bar)	Ref.
Ag	PVDF	35	130 → 150	13 → 17.5	-	[109]
		100		12 → 15	-	
SiO_2_	PVDF	-	143 → 155	10 → 13.4	2.2 → 1.6	[79]
SiO_2_	PVDF	35	124 → 174	6 → 17	2.7 → 5.5	[13]
ZnO	PVDF	12–13	59 → 57	16 → 25	5 → 6	[97]
ZnO	PVDF	38	136 → 140	4 → 9.42	0.7 → 0.4	[65]
TiO_2_	PVDF	100	113.8 → 144	0.5 → 2	-	[34]
CaCO_3_	PVDF	35	72 → 81	35 → 49.4	11 → 8.9	[101]

**Table 7:** Metal-based and oxide nanoparticle performance trade-offs.

Nanoparticle	Key Benefits	Key Drawbacks	Optimal Loading
AgNPs	Antibacterial High CA	Leaching Flux reduction	0.5–1 wt% with PDA [56]
TiO_2_	Photocatalytic Thermal stability	Hydrophilicity High cost	1–2 wt% (hybrid) [96]
ZnO	Non-toxic Antibacterial	Agglomeration LEP reduction	≤0.5 wt% [97]
SiO_2_	High flux Superhydrophobicity	Mechanical fragility	5–7 wt% with MWCNTs [89]
Cu/Ca/Co	Low cost Porosity enhancement	Hydrophilicity Fux variability	-

**Table 8:** Biodegradable nanoparticle key points.

Nanoparticle	Source	Performance in MD
Chitosan	Crustacean shells	Requires hydrophobic additives (e.g., metal oxides) to improve MD performance
Nanocellulose	Plants, algae, bacteria	Effective in RO/NF/MF Limited MD use unless modified
PHA	Microbial synthesis	Not yet viable for MD Weight loss issues in long-term use
PDA	Synthetic (mussel-inspired)	Best MD performance (high flux, low wetting) Sustainability challenges

## Data Availability

The original contributions presented in this study are included in the article. Further inquiries can be directed to the corresponding authors.
